# Estimating the Economic Loss Due to Vibriosis in Net-Cage Cultured Asian Seabass (*Lates calcarifer*): Evidence From the East Coast of Peninsular Malaysia

**DOI:** 10.3389/fvets.2021.644009

**Published:** 2021-10-08

**Authors:** Siti Hajar Mohd Yazid, Hassan Mohd Daud, Mohammad Noor Amal Azmai, Nurliyana Mohamad, Norhariani Mohd Nor

**Affiliations:** ^1^Department of Veterinary Preclinical Sciences, Faculty of Veterinary Medicine, Universiti Putra Malaysia, Serdang, Malaysia; ^2^Department of Veterinary Clinical Studies, Faculty of Veterinary Medicine, Universiti Putra Malaysia, Serdang, Malaysia; ^3^Aquatic Animal Health and Therapeutics Laboratory, Institute of Bioscience, Universiti Putra Malaysia, Selangor, Malaysia; ^4^Department of Biology, Faculty of Science, Universiti Putra Malaysia, Serdang, Malaysia; ^5^Department of Aquaculture, Faculty of Agriculture, Universiti Putra Malaysia, Serdang, Malaysia

**Keywords:** Asian seabass, net-cage culture, vibriosis, economic loss, stochastic model

## Abstract

This study aims to estimate the economic loss due to vibriosis in the production of Asian seabass in floating net-cages on the east coast of Peninsular Malaysia. Asian seabass has contributed significantly to Malaysia's economic activities and food security. However, its production can be hindered by the occurrence of diseases, such as vibriosis, causing severe economic losses to farmers. A questionnaire-based survey was conducted on 14 small-scale monoculture Asian seabass net-cage farms. Using a stochastic bioeconomic model and inputs from the survey, existing literature, and expert opinion, the economic losses were determined. Moreover, this model considered the prevalence of *Vibrio* spp. at a farm on the east coast and the risk posed by its infection from hatcheries. The results showed that 71.09% of Asian seabass simulated in the stochastic model survived. The mortality rate due to vibriosis and other causes was at 16.23 and 12.68%, respectively. The risk posed by *Vibrio* spp. infection from hatcheries contributed to 2.77% of the increase in Asian seabass mortality. The stochastic model estimated that the total cost of producing a tail of Asian seabass was €2.69 per kilogram. The economic loss of vibriosis was estimated at €0.19 per tail per kilogram, which represents 7.06% of the total production cost of Asian seabass per kilogram. An increase in the prevalence of clinical vibriosis and vibriosis case fatality rate at 42 and 100%, respectively, will lead to an increase in the cost of grow-out Asian seabass by €0.29 per tail from the default value. An increase in pellet price per kilogram by €1.38 and feed conversion ratio pellet by 0.96 will consequently increase the cost of grow-out Asian seabass by €2.29 per tail and €0.82 per tail, respectively. We find that the occurrence of *Vibrio* spp. infection at the hatchery level can contribute to an increased risk in the mortality of Asian seabass during the grow-out phase. Hence, we also need to focus on the control and prevention of vibriosis infection from hatcheries.

## Introduction

Asian seabass (*Lates calcarifer*, Bloch 1790) is a euryhaline fish species that tolerates culture crowding and a wide physiological tolerance (1). Asian seabass culture was initiated in Thailand during the early 1970s and expanded to its neighboring countries, such as Indonesia, the Philippines, Singapore, Taiwan, Vietnam, and Malaysia, between the 1980s and 1990s (1). In Malaysia, Asian seabass is commonly cultured in floating net-cages, ponds, tanks, and enclosures (2–4). Its grow-out phase in floating net-cages varies, depending on the final market size and location of grow-out. The grow-out phase for a fingerling size of 6.35 cm during stocking varies between 6 months for 0.7 kg of fish during harvest and 30 months for 3.5 kg of fish suitable for fileting (5, 6). As a carnivorous species, Asian seabass requires a diet with high protein content for its efficient growth. In Malaysia, Asian seabass is fed on commercially formulated feed and trash fish. While the average feed conversion ratios (FCRs) for Asian seabass is ~4 and above for trash fish, they range between 1.5 and 2.1 for commercially formulated feed (7).

Animal diseases can affect the aquaculture production function by destroying basic resources, reducing the physical output or unit value of a production process, lowering the efficiency of a production process, and directly affect human well-being (8, 9); this can ultimately lead to economic losses in the aquaculture sector. Several viral, fungal, parasitic, and bacterial diseases have been reported to affect cage-cultured Asian seabass, which can further cause co-infections (10, 11). In Malaysia, brackish water aquaculture includes the production of Asian seabass, which accounted for 290,900 metric tons in 2018; however, it was a 10.3% decrease from its previous year's production (12). One of the primary factors leading to the fall in production has been attributed to the occurrence of infectious diseases (13). In this context, we focus on vibriosis, a common bacterial disease found in cage-cultured Asian seabass (11, 14). Some members of the genus *Vibrio* spp., such as *V. harveyi, V. alginolyticus*, and *V. vulnificus*, are associated with infections in fish, where the host exhibits clinical signs, such as skin ulceration, scale drops on the abdomen, and necrosis of the caudal fin (9, 14).

However, there exists little information regarding the economic losses caused by vibriosis in the Asian seabass cultured in floating net-cages. In the case of Asian shrimp culture, vibriosis has been reported to have caused losses of USD 1 billion (15). In 1978, loss due to vibriosis in cultured yellowtail (*Seriola quinqueradiata*) was estimated at USD 4.4 million in Japan (16). In the Chinese aquaculture industry, the *Vibrio* spp. infection contributed to a loss of USD 120 million in the early 1990s, where *V. fluvialis* was one of the main pathogens (17). In the early 1990s, outbreaks of *V. harveyi* in the shrimp hatcheries of Indonesia caused economic losses of more than USD 100 million (18). Furthermore, vibriosis was reported to have affected cultured marine fish in Malaysia, causing a loss of USD 7.4 million during the same period (19, 20). Recently, the costs of endemic vibriosis, including treatment and diagnosis costs, for an Asian seabass floating net-cage on the west coast of Peninsular Malaysia was estimated at USD 0.24 per tail (6); however, existing literature has not yet determined the economic loss resulting from vibriosis on the east coast of the country, which is an important area for marine aquaculture. Moreover, small subsistence cage-cultured farms usually do not adopt preventive measures for fingerlings brought from hatchery. Analyzing the risks posed by *Vibrio* spp. from the hatchery may improve our understanding of the influence of vibriosis during the grow-out phase. Consequently, it may improve farmers' awareness of the impact of diseases on production costs and thereby making better decision to reduce the economic losses.

This study aims to fill this research gap by examining the economic loss resulting from vibriosis in the production of Asian seabass in floating net-cages on the east coast of Peninsular Malaysia with the help of a stochastic bioeconomic model (6). Modeling is a useful tool in epidemiology for investigating diseases when experiments and field observations are impracticable (21). Since the bioeconomic model used in this study is stochastic, it enables us to introduce uncertainty in disease prevalence, estimate the losses due to diseases, and observe the consequences of various control strategies, such as fish vaccination, that can be adopted in the future. Similar models have been used in previous studies related to aquaculture and dairy young stock (22).

## Materials and Methods

### The Model

**Figure 1:**
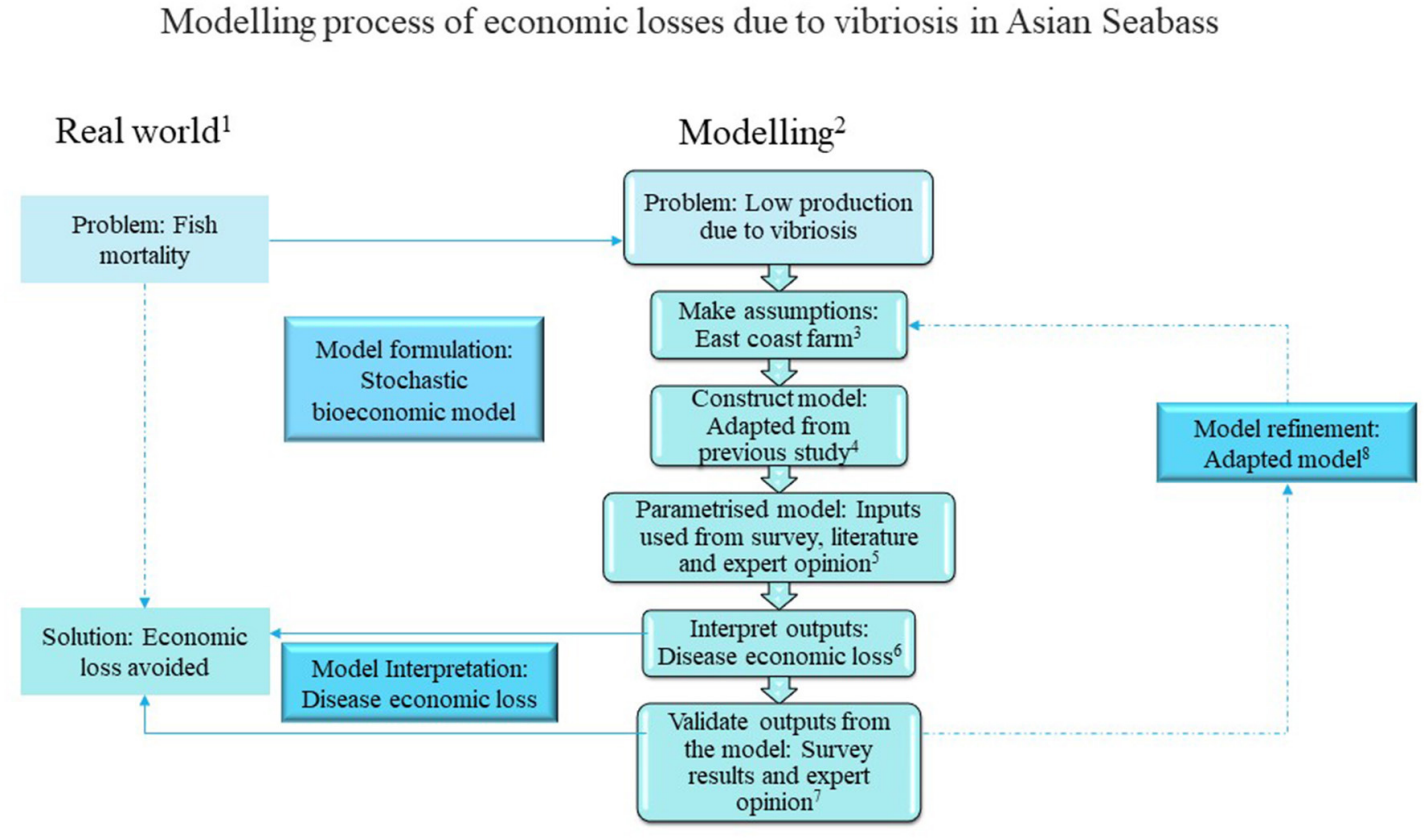
A framework for the estimation of economic losses due to vibriosis in Asian seabass in floating net-cages on the east coast of Peninsular Malaysia. ^1^In the real world, fish mortality is one of the major problems experienced by fish farms, which causes economic losses. A stochastic bioeconomic model is formulated to simulate this issue. ^2^Bioeconomic modeling is used to simulate the problem of low production due to vibriosis: first, we state the relevant assumptions; second, we construct the model in Microsoft Excel using @Risk add-on; third, we select the suitable parameters of the model using biological and economic inputs from existing literature, survey results, and expert opinion; fourth, we interpret and validate the outputs to modify the model where necessary. ^3^Following our assumptions, the bioeconomic model considers small-scale farm management on the east coast of Peninsular Malaysia; we conduct a survey that may help in model formulation. ^4^We adapt the bioeconomic model from that of a previous study based on Asian seabass grow-out in cage farms on the west coast of Peninsular Malaysia. Details of the modeling of economic losses are shown in Figure 2 and Supplementary Figure 1. ^5^We obtain biological and economic inputs by conducting farm surveys, analyzing the prevalence of vibriosis from fish sampling, existing literature, and expert opinion (see Tables 1–5). ^6^We interpret the model output in terms of economic losses from vibriosis (see section Analysis of the economic loss due to vibriosis using a stochastic model). ^7^Survey results (see section Survey results) and expert opinion are used to validate the model output. ^8^We modify the model by adapting small-scale farm management on the east coast of Peninsular Malaysia. This model includes the risk of *Vibrio* spp. infection from hatcheries and grow-out cage culture, assuming that the diagnosis or treatment for infected Asian seabass are not viable (assumption in the model refers to the inputs used in Tables 1–5).

Following a prior study (6), we employed a stochastic bioeconomic model built in Microsoft Excel® (Microsoft Corp., Redmond, WA, USA) using @Risk add-on (Palisade Corp., Ithaca, NY, USA) to estimate the economic losses due to vibriosis on the east coast of Peninsular Malaysia. Disease prevalence on the east coast could differ from that on the west coast because of the differences in their environmental characteristics, including the physicochemical parameters of water (10, 11). Figure 1 presents the framework for the estimation of the economic loss due to vibriosis in Asian seabass in floating net-cages on the east coast of Peninsular Malaysia (23, 24). The economic and biological inputs obtained in this study were based on a survey conducted on the east coast (section Model inputs), existing literature, and expert opinion.

Our stochastic bioeconomic model comprised a total of 18 two-weekly stages that helped in determining the health status of Asian seabass. Moreover, it considered the risk posed by *Vibrio* spp. infection in hatcheries, as a result of farmers' inability to adopt preventive measures when cultivating fingerlings to grow-out cage culture. This model estimated the cost of grow-out from a body weight of 21 g to 1 kg within 210 days. Supplementary Figure 1 in the Supplementary materials section presents the stochastic model that simulated the costs of the infected during the grow-out phase, and dead Asian seabass due to vibriosis. Our model assumed small-scale farm management on the east coast, where farm owners did not provide treatment to the infected or send diseased fish samples in the laboratory for diagnosis; therefore, there was no estimation of diagnosis and treatment cost for infected Asian seabass. The economic loss of vibriosis was estimated by the sum of variable costs to grow-out fish that died due to vibriosis divided by the number of Asian seabass that survived until market age (refer to Supplementary Equation 1 in the Supplementary materials section). The model was simulated by 10,000 iterations, considering the currency exchange as €1= RM 4.94, on February 25, 2021.

**Table 1:** Data on the prevalence of subclinical and clinical *Vibrio* spp.

**Month**	**Prevalence of subclinical *Vibrio* spp.^a^^b^**	**Prevalence of clinical *Vibrio* spp.^c^**
January	0.20	0.17
February	0.32	0.12
March	0	0.12
April	0.60	0.11
May	0.32	0.14
June	0.32	0.24
July	0.60	0.32
August	0.40	0.40^b^
September	0.32	0.28
October	0.25	0.24
November	0.20	0.16
December	0.32	0.42

*Subclinical Vibrio spp. is defined as Asian seabass positive with at least one species of Vibrio that do not exhibit clinical signs. Clinical Vibrio spp. is defined as Asian seabass positive with at least one species of Vibrio, exhibiting either internal or external clinical signs or both*.

a *Constructed following a stochastic bioeconomic model, using Riskpert [minimum, most likely, and maximum prevalence, RiskTruncate (0,1)] due to insufficient data. For example, for January, we have Riskpert [0, 0.20, 0.60, RiskTruncate (0,1)]*.

b *Prevalence of Vibrio spp. at Sungai Marang, Terengganu (unpublished data). Sampling is conducted between October 2018 and August 2019. Vibrio spp. isolated from Asian seabass in this study are V. fluvialis, V. vulnificus, V. alginolyticus, and V. parahaemolyticus*.

c *Prevalence of clinical Vibrio spp. at Pulau Ketam, Selangor (11)*.

**Figure 2:**
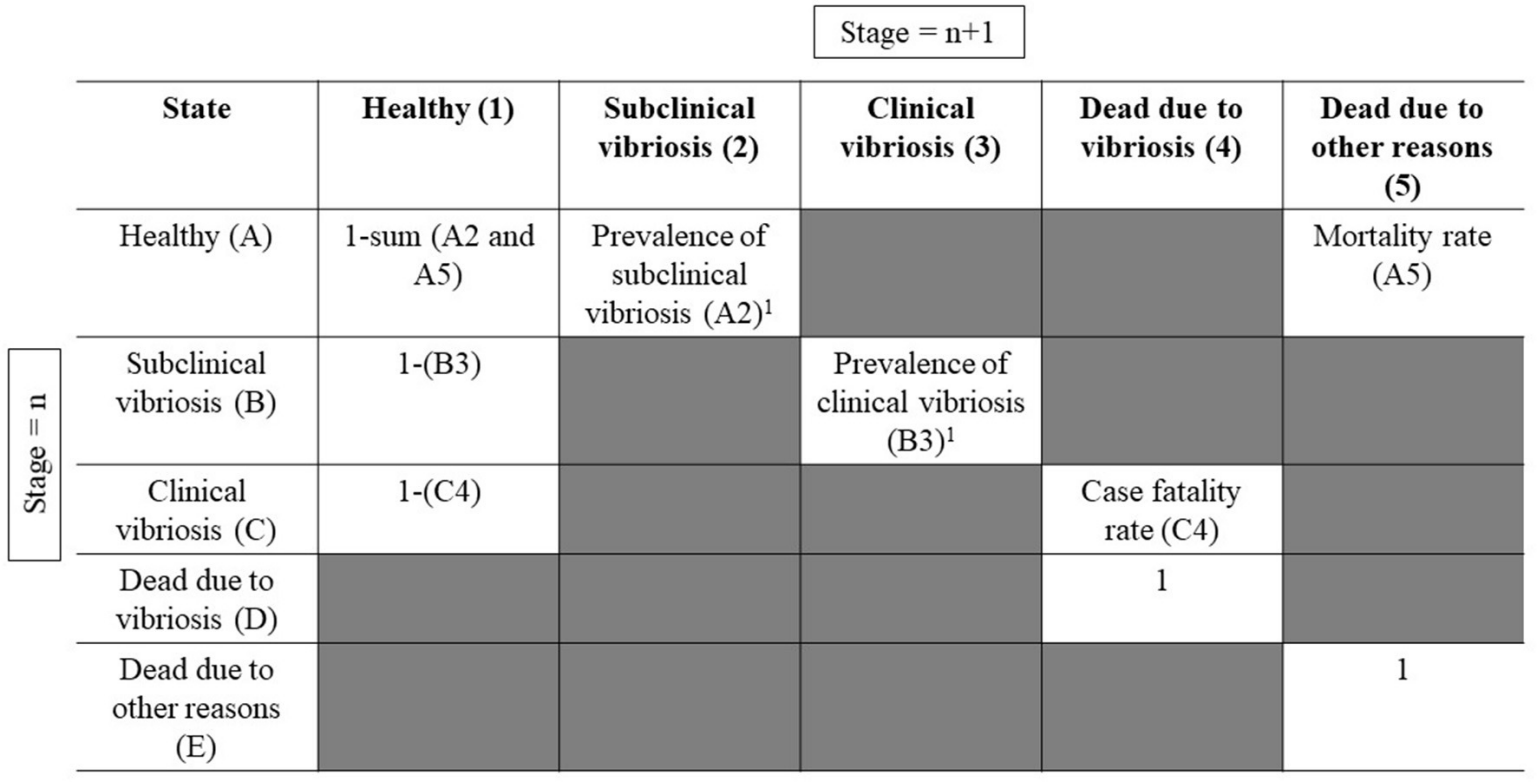
This transition matrix was adapted from a previous study (6). The state of fish at stage (*n* + 1) is dependent on its state at the previous stage (*n*). From the figure above, we can see that at stage *n*, a fish in a healthy state (cell A) can be subclinically infected with vibriosis (cell 2) at stage (*n* + 1), determined by the prevalence of subclinical vibriosis (A2). Within the following 2 weeks of subclinical infection with vibriosis (cell B), we assume that there will be a response from the fish's immune system, such that it will either become healthy (cell 1) or clinically infected with vibriosis (cell 3), determined by the prevalence of clinical vibriosis (B3). Within 2 weeks after acquiring a clinical infection with vibriosis (cell C), a fish can either become healthy (cell 1) or die (cell 4), which is determined by the case fatality rate (C4). If the state of the fish implies that it was dead in the previous stage (*n*), it will remain the same in the following stages (D4 and D5). ^1^Indicates differences in the rate of infection, depending on the month of grow-out (11).

A transition matrix was used to determine the health status of Asian seabass at each stage, referred to as a state, as shown in Figure 2. The model considered five states to determine the health status of Asian seabass: healthy, subclinical vibriosis, clinical vibriosis, dead due to vibriosis, and dead due to other causes. This study included only those cases where the fish was positive with at least one type of *Vibrio* sp. and did not display any clinical signs, which is defined as subclinical vibriosis. For clinical vibriosis, the fish must be positive with at least one type of *Vibrio* sp. and show either external or internal clinical signs or both. Inputs from the prevalence of subclinical *Vibrio* spp. infection, the prevalence of clinical *Vibrio* spp. infection, case fatality rate due to *Vibrio* spp., and mortality rate due to other reasons were used to determine the states.

### Model Inputs

The inputs used in this study are based on the output of farm surveys, existing literature, and opinions from Malaysian fish disease experts. The biological input of the stochastic bioeconomic model includes the prevalence of subclinical and clinical *Vibrio* spp. in the east coast region, specifically Marang, Terengganu (unpublished data) (Table 1), the prevalence of subclinical and clinical *Vibrio* spp. in hatcheries (25), the prevalence of clinical *Vibrio* spp. (grow-out) from a fish farm in Pulau Ketam, Malaysia (Table 1) (11), case fatality rate during the grow-out phase with an average of 40% (26), number of deaths in Asian seabass due to other reasons (Table 2) (27), gain in body weight (28), seawater temperature (Table 3) (29), FCR for pellet (1.73–2.96) and trash fish feed (3.53–4.16) (7, 30–32), and feed attribution to gain in body weight (60% pellet; 40% trash fish) (32). Based on previous recommendations, we ensure that the amount of feed consumed per kilogram body weight was not more than 10% of the body weight (33). A summary of the biological inputs used in this study is shown in Table 4. The economic input of the stochastic bioeconomic model includes fingerling price (€0.24 per tail), pellet price (€1.26 per kilogram), trash fish price (€0.18 per kilogram), labor cost (€313 per month), maintenance cost (€10 per month), petrol cost (€17.59 per month), and utility cost (€4.99 per month) based on the output of the surveys conducted (Table 5).

**Table 2:** The number of dead fish per stage is modeled using Riskpert (minimum, most likely, and the maximum number of dead).

**Grow-out stage**	**Post-stocking (days)**	**Minimum number of dead**	**Most likely number of dead**	**Maximum number of dead**
1	1	210	350	1,092
2	14	210	350	1,092
3	28	210	350	1,092
4	42	70	140	280
5	56	42	112	210
6	70	28	70	126
7	84	0	56	182
8	98	0	56	182
9	112	14^a^	56	182
10	126	14	70	182
11	140	14	70	182
12	154	14	70	182
13	168	14	70	182
14	182	14	70	182
15	196	14	70	182
16	210	14	70	182

*For example, we observe Riskpert (0, 300, 1,500) due to uncertainty in mortality based on previous data (27)*.

a *The minimum number of dead increases after 112 days post-stocking, as the weight of Asian Sea bass has reached 400 g that is the market weight under grading*.

**Table 3:** Seawater temperature on the east coast of Peninsular Malaysia (29).

**Month**	**Average temperature (^**°**^C)**	**Standard deviation**
January	27.05	0.25
February	27.47	0.23
March	28.12	0.17
April	29.25	0.12
May	29.93	0.22
June	29.90	0.17
July	29.70	0.17
August	29.25	0.12
September	29.23	0.05
October	29.55	0.05
November	29.15	0.05
December	28.10	0.13

*The data are used in the stochastic bioeconomic model to estimate the gain in body weight (28) by using a normal distribution [RiskNormal (average, standard deviation)]. For example, for January, we have RiskNormal (27.05, 0.25)*.

**Table 4:** Other biological inputs used in the stochastic bioeconomic model for grow-out in Asian seabass.

**Variable**	**Data**	**Sources**
**Percentage of gain in body weight attributed to feed**		
Before 2 months of age	100% by pellet	[Farm survey, (32)]
Between 2 and 4 months of age	80% by pellet; 20% by trash fish	[Farm survey, (32)]
After 4 months of age	60% by pellet: 40% by trash fish	(32)
**The feeding rate per day**		
Before fingerlings attain a body weight of 100 g	3	[Farm survey, (30, 33)]
After fingerlings attain a body weight of 100 g	2	Farm survey
The feed conversion ratio for trash fish (average)^a^	1: 4	(7, 30, 32)
Minimum–maximum	3.53–4.16	
The feed conversion ratio for pellet (average)^a^	1:2	(7, 31, 32)
Minimum–maximum	1.73–2.96	
**Vibriosis**		
Prevalence of subclinical *Vibrio* spp. in hatcheries^a^	0.077	(25)
Prevalence of clinical *Vibrio* spp. in hatcheries^a^	0.083	(25)
Case fatality rate (minimum–maximum)^a^	40% (0%−100%)	(25)
**Body weight gain constants**		
K	2.2495	(28)
X	−0.327	
Y	0.015	
Z	−0.000203	
a	−0.01	
b	0.72	
Body weight loss (*Vibrio* spp.)	0.69% from body weight	(11)
**Time taken to do activities**		
Cleaning net (seconds per tail)	2.7	Aquatic veterinarian
Feeding (seconds per tail)	0.8	Aquatic veterinarian
Grading (seconds per tail)	1.8	Aquatic veterinarian

a *Modeled using Riskpert (minimum, most likely, and maximum number). An example of the case fatality rate is Riskpert (0, 0.40, 1)*.

**Table 5:** Economic inputs used in the stochastic model.

**Variables**	**Price (€)**	**Source**
Fingerling at 3 to 4 inches (per tail)	0.24	Farm survey
Market fish (per kilogram)	3.29	Farm survey
Pellet (per kilogram)	1.26	Farm survey
Trash fish (per kilogram)	0.18	Farm survey
Labor wage (per month)	313	Farm survey
Maintenance (per month)	10	Farm survey
Petrol (per month)	17.59	Farm survey
Utility (per month)	4.99	Farm survey

#### Farm Survey

Malaysia is divided into Peninsular Malaysia and Borneo Island. Peninsular Malaysia is divided into the east coast and west coast. The east coast of Peninsular Malaysia consists of three states: Kelantan, Terengganu, and Pahang. A list of 209 floating net-cage farms in the three states was obtained from the Department of Fisheries (DOF), Ministry of Agriculture and Agro-food Industry, Malaysia. Using convenience sampling, we selected 39 farms to be surveyed between February and May 2017. During the survey, a face-to-face interview was conducted with the farm owner or representative of the workers at the farm with the help of a questionnaire. The questionnaire used in this study contains 122 questions, consisting of five sections that include questions on farmers' backgrounds, farm management and background, general fish health information, and knowledge of vibriosis with reference to previous studies (6). The data collected were based on the latest fish culture cycle of 2016.

### Model Validation

The survey results were used to validate the model output for feed. Furthermore, the opinions of Malaysian fish disease experts were used to validate the model output for the mortality rate due to vibriosis.

### Sensitivity Analysis

Sensitivity analyses were conducted on crucial economic and biological inputs to determine the impact of a change in input on the costs of grow-out in Asian seabass per kilogram per tail. The default input value was changed one at a time to a lower or higher value. The following economic inputs were changed: (i) fingerling price per tail with default a value at €0.24 was changed one at a time to €0.20 (– €0.04 from default value) and further changed to €0.32 (+ €0.06 from default value); (ii) trash fish price per kilogram with a default value at €0.18 (– €0.07; + €0.53); (iii) pellet price per kilogram with a default value at €1.26 (– €0.46; + €1.38); (iv) labor cost per month with a default value at €313 (– €70; + €70). The following biological inputs were changed: (i) FCR pellet with a default value most likely at 2 (−0.27; +0.96) (7, 31, 32); (ii) FCR trash fish with a default value most likely at 4 (−0.47; +0.16) (7, 30, 32); (iii) prevalence of subclinical *Vibrio* spp. during grow-out phase (0%, 60%) (unpublished data); (iv) prevalence of clinical *Vibrio* spp. during grow-out phase (11%, 40%) (11); (v) *Vibrio* spp. case fatality during grow-out phase with a default value most likely at 40% (−40%, +60%) (26) (Table 6).

**Table 6:** Sensitivity analyses for biological and economic inputs.

**Variables**	**Default value (€)**	**Change in value (lowest change; highest change) (€)**	**Source**
Fingerling	0.24	−0.04; +0.06	Farm survey
Trash fish (per kilogram)	0.18	−0.07; +0.53	Farm survey
Pellet price (per kilogram)	1.26	−0.46; +1.38	Farm survey
Labor wage (per month)	313	−70; +70	Farm survey
Feed conversion ratio (pellet)	2	−0.27; +0.96	(7, 31, 32)
Feed conversion ratio (trash fish)	4	−0.47; +0.16	(7, 30, 32)
Prevalence of subclinical vibriosis	Refer to Table 1	(−0 to −0.60); (+0 to +0.60)	Unpublished data
Prevalence of clinical vibriosis	Refer to Table 1	(−0 to −0.31); (+0; to +0.31)	(11)
Vibriosis case fatality rate	0.40	−0.40; +0.60	(26)

### Data Management and Analysis

Data collected from the survey were inserted and edited using Microsoft Excel® (Microsoft Corp. Inc, Ithaca). We conducted a descriptive analysis using R-version 3.3.1 (R Foundation for Statistical Computing; Vienna, Austria). Additionally, we conducted a descriptive analysis on the output of the stochastic model using the StatTools add-on (Palisade Corp. Ithaca, NY, USA) in Microsoft Excel® (Microsoft Corp. Redmond, WA, USA).

## Results

### Analysis of the Economic Loss Due to Vibriosis Using a Stochastic Model

Based on our stochastic model, 71.09% of the simulated Asian seabass survived for 210 days post-stocking with an average body weight of 1,060 g (5–95% percentiles: 1,045–1,075 g). A total of 1,494 g of commercially formulated feed and 1,317 g of trash fish were consumed per tail. The mortality rate due to vibriosis and other causes was 16.23 and 12.68%, respectively. The total average cost of producing a tail of Asian seabass was €2.69, consisting of €2.36 variable costs, €0.02 fixed costs, and €0.31 provision costs due to mortality (Supplementary Equation 21 in the Supplementary materials section). The total economic loss due to vibriosis was estimated at €0.19 per tail (Table 7), representing 7.06% of the total production cost of Asian seabass per kilogram (Supplementary Equation 22 in the Supplementary materials section).

**Table 7:** The cost of grow-out for Asian seabass in cage culture per tail from 20 to 1,060 g (5–95% percentiles: 1,045–1,075 g) within 210 days of using the stochastic model.

**Variables**	**Average cost (5%−95% percentiles) (€)**	**Type of costs**	**Average cost (5%−95% percentiles) (€)**
Fingerling	0.24	Variable^a^	0.24
Feed			2.11 (1.87–2.41)
Trash fish	0.23 (0.22–0.24)		
Pellet	1.87 (1.64–2.18)		
Labor	0.012 (0.012–0.012)		0.012 (0.012–0.012)
Total			2.36 (2.12–2.66)
Maintenance	0.008 (0.008–0.008)	Fixed^a^	
Petrol	0.012 (0.012–0.012)		
Utility	0.004 (0.004–0.004)		
Total			0.02 (0.02–0.02)
Losses (total mortality)		Provision^a^	0.31
Due to vibriosis	0.19		
Due to other cause	0.12		
Variables	Average cost (5%−95% percentiles) (€)	Type of profit^a^	Profit
Fish (g)	1,060 [1,045–1,075]	Revenue	3.49 (3.44–3.54)
		Gross margin	1.12 (0.83–1.37)
		Net profit	1.10 (0.72–1.26)

a *Refer to Supplementary Equations 11–20 in the Supplementary materials*.

**Figure 3:**
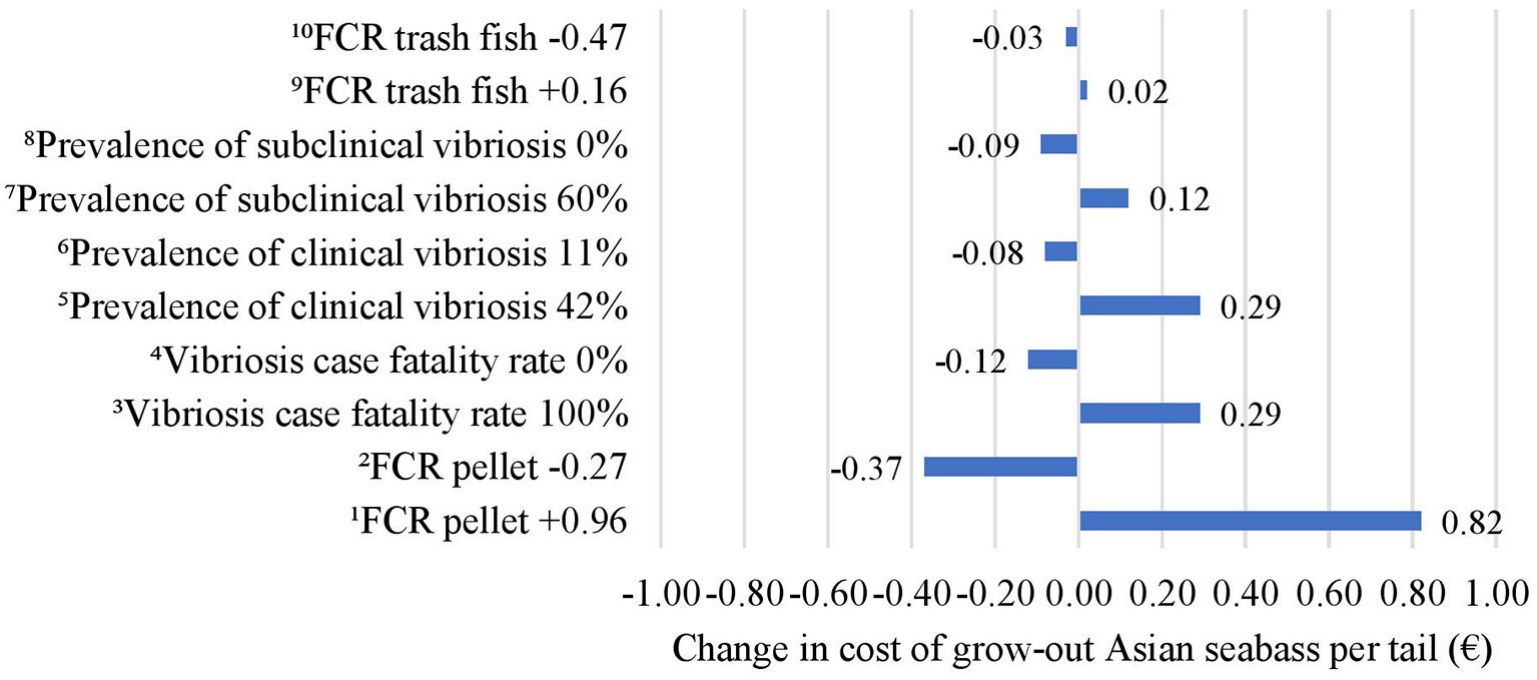
Impact of change in biological inputs on the costs of grow-out in Asian seabass per tail. ^1^When the FCR for pellet increases by 0.96 from its default value, the costs of grow-out increase by €0.82 per tail. ^2^When the FCR for pellet decreases by 0.27 from its default value, the costs of grow-out decrease by €0.37 per tail. ^3^When the case fatality rate due to vibriosis is 100%, the costs of grow-out increase by €0.29 per tail. ^4^When the case fatality rate due to vibriosis is 0%, the costs of grow-out decrease by €0.12 per tail. ^5^When the prevalence of clinical vibriosis is 42%, the costs of grow-out increase by €0.29 per tail. ^6^When the prevalence of clinical vibriosis is 11%, the costs of grow-out decrease by €0.08 per tail. ^7^When the prevalence of subclinical vibriosis is at 60%, the costs of grow-out increase by €0.12 per tail. ^8^When the prevalence of subclinical vibriosis is at 0%, the costs of grow-out decrease by €0.09 per tail. ^9^When FCR for trash fish increases by 0.16, the costs of grow-out Asian seabass increase by €0.02 per tail. ^10^When FCR for trash fish decreases by 0.47, the costs of grow-out decrease by €0.03 per tail.

Sensitivity analysis conducted on biological inputs showed that the costs of grow-out in Asian seabass per tail were most sensitive to changes in the FCR for pellet and case fatality rate due to vibriosis. When the FCR for pellet increased by 0.96 from its default value, the costs of grow-out in Asian seabass increased by €0.82 per tail. When the FCR for pellet decreased by 0.27 from its default value, the costs of grow-out decreased by €0.37 per tail. Since the case fatality rate due to vibriosis increased to 60% from its default value (40%), the costs of grow-out increased by €0.29 per tail. When the case fatality rate due to vibriosis was reduced to 0%, the costs of grow-out decreased by €0.12 per tail. When the prevalence of clinical vibriosis was at 42%, the costs of grow-out increased by €0.29 per tail. When the prevalence of clinical vibriosis was at 11%, the costs of grow-out decreased by €0.08 per tail. When the prevalence of subclinical vibriosis increased to 60%, the costs of grow-out increased by €0.12 per tail. When the prevalence of subclinical vibriosis was reduced to 0%, the costs of grow-out decreased by €0.09 per tail. When the FCR for trash fish increased by 0.16, the costs of grow-out Asian seabass increased by €0.02 per tail. When FCR for trash fish decreased by 0.47, the costs of grow-out decreased by €0.03 per tail (Figure 3).

**Figure 4:**
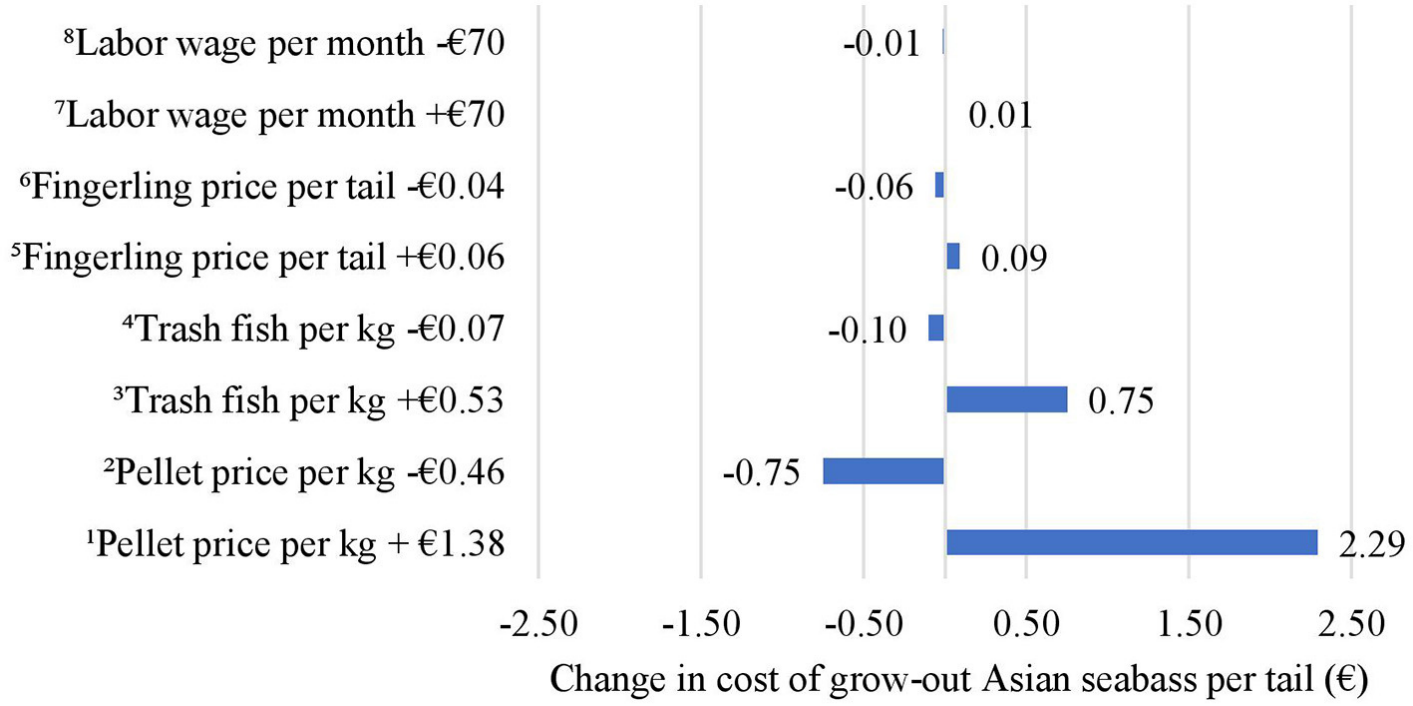
Impact of changes in economic inputs on the costs of grow-out in Asian seabass per tail. ^1^When the price of pellet per kilogram increases by €1.38 from its default price, the costs of grow-out increase by €2.29 per tail. ^2^When the price of pellet per kilogram decreases by €0.46 from its default price, the costs of grow-out decrease by €0.75 per tail. ^3^When the price of trash fish per kilogram increases by €0.53, the costs of grow-out increase by €0.75 per tail. ^4^When the price of trash fish per kilogram decreases by €0.07 less, the costs of grow-out decrease by €0.10 per tail. ^5^When the fingerling price per tail increases by €0.06, the costs of grow-out increase by €0.09 per tail. ^6^When the fingerling price per tail decreases by €0.04, the costs of grow-out decrease by €0.06 per tail. ^7^When the wage of labor per month increases by €70, the costs of grow-out increase by €0.01 per tail. ^8^When the wage of labor per month decreases by €70, the costs of grow-out decrease by €0.01 per tail.

Based on the sensitivity analysis conducted on economic inputs, when the price of pellet per kilogram increased by €1.38 from its default price of €1.26, the costs of grow-out increased by €2.29 per tail. When the price of pellet per kilogram decreased by €0.46 from its default price, the costs of grow-out decreased by €0.75 per tail. Similarly, an increase in the price of trash fish per kilogram by €0.53 increased the costs of grow-out by €0.75 per tail. When the price of trash fish per kilogram decreased by €0.07, the costs of grow-out decreased by €0.10 per tail. An increase in the fingerling price per tail by €0.06 increased the costs of grow-out by €0.09 per tail. A decrease in the fingerling price per tail by €0.04 decreased the costs of grow-out by €0.06 per tail. An increase in the wage of labor per month by €70 increased the costs of grow-out by €0.01 per tail and vice versa (Figure 4).

### Survey Results

The average age of the farmers was 54 years (minimum–maximum: 37–70 years). All the observed farmers (*n* = 14) were male with the highest level of educational attainment being secondary education (*n* = 8). We observed that the farmers (*n* = 14) did not know about the vibriosis disease, including its clinical signs. However, majority of the farmers attributed the reasons for sick and dead fish to both infectious and non-infectious agents (*n* = 10), and non-infectious agents only (*n* = 4). The median morbidity rate at the farms was perceived as 50% per cycle (minimum–maximum: 17.5%−80%), while the median mortality rate was perceived as 50% per cycle (minimum–maximum: 17.5%−80%). Although 50% of the farmers (*n* = 7) provided treatment using freshwater, the remaining did not resort to any type of treatment (*n* = 7). Majority of the farmers (85%, *n* = 6) reported that none of the fish recovered after treatment.

The median of Asian seabass net-cage per farm (*n* = 14) was 13 cages (minimum–maximum: 6–32 cages) with a median cage size of 9.30 m^2^ (minimum–maximum: 7.44–18.57 m^2^). The median stocking density per square meter of a net-cage was 67 tails (minimum–maximum: 18–107 tails). The median stocking density per farm (*n* = 14) was 3,000 tails (minimum–maximum: 1,000–12,000 tails) with a stocking fingerling size of 10.16 cm (minimum–maximum: 6.35–27.94 cm) (*n* = 14). The median grading frequency at the farms was three times per cycle (minimum–maximum: 1–4 times per cycle). The median grow-out period per cycle (*n* = 14) was 12 months (minimum–maximum: 6–24 months) with the median size of fish being 1 kg (minimum–maximum: 0.7–1.5 kg). The median number of Asian seabass harvested per cycle (*n* = 14) was 1,350 tails (minimum–maximum: 400–4,800 tails). The median tonnage of Asian seabass harvested per cycle (*n* = 14) was 1.05 metric tons (minimum–maximum: 0.34–4.8 metric tons). On average, the feeding frequency at the farms surveyed was twice per day in the first month of culture, which was consequently reduced to one and two times per day. None of the surveyed farms were accredited with the Malaysia Good Aquaculture Practices (MyGAP) certification.

On average, following the monthly operational costs incurred by the surveyed farms, the highest cost was attributed to commercially formulated feed (78.7%), followed by trash fish (16.33%), fuel (2.64%), and maintenance (1.56%). The least amount of expenditure was attributed to utility (0.77%) (Table 8). The estimated median cost of producing 1,350 tails per kilogram of Asian seabass was €3,288 per cycle. The median cost of fingerlings (*n* = 14) was €728 per cycle. The median cost of feed (*n* = 14) was €2,281. The median expenditure for labor (*n* = 14), maintenance (*n* = 9), fuel (*n* = 14), and utility (*n* = 14) were €0 per cycle.

**Table 8:** Descriptive results of continuous data on operational costs per month.

**Variable**	**Median (€)**	**Mean (€)**	**Minimum (€)**	**Maximum (€)**	**Number of farms (*n* = 14)**
**Monthly estimation^a^**					
Commercially formulated feed	264	506	78	2,370	14
Trash fish	94	105	0	288	8
Petrol per diesel	0	17	0	200	13
Maintenance (e.g., net and cage repair)	0	10	0	90	9
Utility (electricity)	0	4.93	0	48	14

a *The median grow-out period per cycle at survey farms (n = 14) was 12 months (minimum–maximum: 6–24 months)*.

## Discussion

According to our model, the estimated economic loss due to vibriosis is €0.19 per tail, representing 7.06% of the total production cost of Asian seabass per kilogram. A previous study reported the cost of endemic vibriosis to be €0.004 higher than the results reported in our study since the study included diagnosis and treatment costs (6). Loss due to vibriosis in this study could be higher than the previous study if the diagnosis and treatment were included due to the risk of *Vibrio* spp. infection from hatcheries and higher prevalence of *Vibrio* spp. on the east coast. Studies on the costs and benefits of vibriosis control and prevention through vaccination in hatcheries and constructing a better grow-out net-cage farms on the east coast should be taken into consideration. In this study, the surveyed farmers estimated their production as 1,350 tails of 1 kg of Asian seabass per cycle. For example, considering the analysis of the cage culture area and the costs of vibriosis using model in this study, there could be provisional costs estimated at €265 (8.06%) in addition to the total cost of €3,288 per cycle. Analysis in this study revealed that 16.23% of simulated Asian seabass died due to vibriosis, which is in contrast to earlier findings that reported a 6.89% mortality rate due to vibriosis from the stochastic model (6). This could be due to the differences in the occurrence of vibriosis in the observed farms. Floating net-cage farms located in the east coast area are prone to flooding because of the annual northeast monsoon that causes fluctuations in water physicochemical parameters. All the floating net-cage farms observed in this study were situated close to the land; in addition, the river is known for low water tidal current episodes that cause river water to become stagnant, inevitably providing favorable conditions for the growth of *Vibrio* spp. Following the sensitivity analysis, other than the feed that greatly influences the costs of grow-out in Asian seabass per tail, disease such as high rate of *Vibrio* spp. infection, particularly fatality rate due to vibriosis and prevalence of clinical vibriosis, highly influences the costs of grow-out in Asian seabass. An increase in vibriosis case fatality rate and the prevalence of clinical vibriosis at a maximum rate of 100 and 42%, respectively, would increase the costs of grow-out by €0.29 more per tail from its default value, thereby increasing the costs by 12%. If measures of controlling the occurrences of diseases at farms are not taken, it can cause significant losses, resulting in a high cost of grow-out in Asian seabass per tail. An increase in the production costs due to disease could be overcome by adopting suitable biosecurity measures at farms, such as minimal handling of fish (e.g., during fish stocking and grading), appropriate disposal of dead fish, quarantine of sick fish, use of appropriate feeds, and regularly conducting laboratory analyses to check the status of fish health. In addition, implementation of vaccination and chemoprophylaxis as preventive measures should be considered to mitigate economic losses (33, 34).

This study presented the results of a survey of 14 small-scale monoculture Asian seabass farms in the east coast of Malaysia based on the information provided by its Department of Fisheries. Despite the small sample size, the study conducted surveys on cage culture farms in the states of Kelantan and Terengganu, considering different farm sizes and management inputs, which can provide insights into small-holder cage culture farms. Our study showed that the market price of Asian seabass in the east coast was higher by €0.19 as compared to the west coast (6), which could be a result of a less fish supply in the east coast. Moreover, production in the east coast could be affected by the yearly harsh monsoon season and Asian seabass on the east coast is mostly marketed to the west coast. It should be noted that the total cost of grow-out for the tail of Asian seabass was estimated at €2.69 in this study, which is €0.28 lower than that on the west coast (6). In addition to the low cost of trash fish, we observed that all cage culture sites were close to land; thus, the farmers did not have to spend significantly on fuel and maintenance costs in comparison to the west coast region of Peninsular Malaysia (6). We found that the net profit obtained from the east coast farm was €0.44 per tail higher than that in the west coast (6).

In this study, we found that the risks posed by *Vibrio* infection from hatcheries contributed to 2.77% of the increase in Asian seabass mortality due to vibriosis. A previous study on marine fish fry, that is Asian seabass, red snapper, and hybrid grouper, reported the prevalence of *Vibrio* spp. in more than half (55%) of the fish sampled (25). It was stated that the major source of infection by pathogens could be transmitted through the feed at the hatchery (25, 35), and *Vibrio* spp. were introduced to the grow-out cages by an infected fry once they were transferred. The transfer of fingerlings must be carried out cautiously to overcome economic losses. It is recommended for the farmers to stock high-quality fingerlings obtained from reputable suppliers and are free from diseases. The transport used to deliver the fingerlings from hatcheries must be cleaned, rinsed, and disinfected before and after delivery to the farm. In addition, the fingerlings must be quarantined upon their arrival before being stocked into net-cages; this will allow the farmers to observe any prevailing disease infection and help the fish to adapt to its new environment by minimizing its stress associated with the new environment (33).

From the stochastic model, the total variable cost of grow-out per kilogram of Asian seabass was estimated at €2.36 per tail. The findings are consistent with the survey results, which showed that the estimated median cost of grow-out for 1,350 tails of Asian seabass per kilogram was €3,288 per cycle, implying that the cost of producing a tail of Asian seabass with a body weight of 1 kg is €2.43. Previous findings reported the total variable costs of grow-out for Asian seabass per kilogram using a stochastic model to be slightly higher at €2.59 per tail (6). The observed low variable cost estimated in this study could be due to the differences in the prices of feeds. Majority of farmers in the east coast depend on discarded fish heads instead of whole fish as feed since they are readily available with much lower cost. However, these findings need to be interpreted with caution due to the uncertainty associated with the FCR for fish heads, which could be higher than that for a whole fish and could affect the growth rates (7), and a longer grow-out period to reach the market size, consequently increasing the production costs of the farm. To the best of our knowledge, no prior study has reported on the FCR for fish heads; hence, it is suggested that future research needs to focus on this gap. Our study found that commercial pellets were the largest cost component at €1.87 per tail, which was 69% of the total costs of grow-out per tail in Asian seabass. These findings are consistent with previous studies (5–7). In fact, the cost of grow-out in Asian seabass was most sensitive to changes in pellet prices and the FCR for pellet. A decrease in the pellet price by €0.46 from its default value (36% of the price of pellet per kilogram) could reduce the costs of grow-out Asian seabass by €0.75 per tail (31% lower costs) while a lower FCR of pellet by 0.27 from its default value (14% lower FCR) could reduce the cost of grow-out in Asian seabass by €0.37 per tail (16% lower costs). To improve feed and feeding efficiency, future studies should focus on the development of feed quality with an appropriate formulation and stable protein content that is highly digestible by Asian seabass in its different stages of growth and development. Additionally, suitable feeding regimes, feeding protocols, effective feeding systems, and on-farm feed management strategies and technologies should be developed, which can be implemented by farmers.

In conclusion, the stochastic model employed in this study estimated the economic losses due to vibriosis in Asian seabass cage culture in the east coast of Peninsular Malaysia at €0.19 per tail, which represents 7.06% of the total production cost of Asian seabass per kilogram. The total cost of producing a tail of Asian seabass was estimated at €2.69 per kilogram per tail, which comprises variable costs, such as the sum of operational costs at €2.36, fixed costs at €0.02, and provision costs due to mortality at €0.31. The findings from this study provide insights into the economic losses due to vibriosis in the context of cage-cultured Asian seabass kept in the east coast of Peninsular Malaysia. The findings of this study can contribute toward decision-making in the context of the prevention and control of fish diseases. Additionally, this study provides a better understanding on the cost of production of Asian seabass in floating net-cages for farmers and increase their awareness of the impact of such disease on Asian seabass production. Furthermore, this can help the farmers in managing their farm by practicing good farm management, such as proper record-keeping, ensuring the optimal stocking density of fish per net-cage, proper feeding management, regular water quality monitoring, and implementing appropriate biosecurity measures, such as proper equipment sanitation, farm access control, quarantine and treatment of sick fish, and appropriate disposal of dead fish at the farm; consequently, this may improve the economy of the aquaculture industry. The risk associated with *Vibrio* spp. infection from hatcheries has contributed to the mortality of Asian seabass during the grow-out phase. Hence, attention needs to be paid toward the control and prevention in the transmission of *Vibrio* spp. from hatcheries.

## Data Availability Statement

The raw data supporting the conclusions of this article will be made available by the authors, without undue reservation.

## Author Contributions

NMN, SHMY, and HMD: conceptualization. NMN and SHMY: methodology. NMN and HMD: validation. SHMY and NMN: formal analysis. SHMY, NMN, MA, NM, and HMD: data curation. SHMY: original draft preparation. NMN, HMD, and MA: writing, review, and editing. All authors have reviewed and read the manuscript.

## Funding

This study was financially supported by the Ministry of Higher Education, Malaysia under the Transdisciplinary Research Grant Scheme (Project id mygrants: 14872; Reference code: TRGS/1/2019/UPM/02/5/; Vot UPM: 5535902) (Title: Evaluating the effects of vibriosis vaccination on socio-economic of cage culture marine fish industry). SHMY is the recipient for Special Graduate Research Allowance (SGRA) scheme at Universiti Putra Malaysia.

## Conflict of Interest

The authors declare that the research was conducted in the absence of any commercial or financial relationships that could be construed as a potential conflict of interest.

## Publisher's Note

All claims expressed in this article are solely those of the authors and do not necessarily represent those of their affiliated organizations, or those of the publisher, the editors and the reviewers. Any product that may be evaluated in this article, or claim that may be made by its manufacturer, is not guaranteed or endorsed by the publisher.
