# Supplementary material for: Estimating the Economic Loss Due to Vibriosis in Net-Cage Cultured Asian Seabass (Lates calcarifer): Evidence From the East Coast of Peninsular Malaysia
Source: Front Vet Sci. 2021 Oct 8;8:644009. doi: 10.3389/fvets.2021.644009 (PMC8531722; doi:10.3389/fvets.2021.644009)
Supplement: Supplementary file 1 [file Data_Sheet_1.PDF]

Supplementary Material

1 SUPPLEMENTARY FIGURE

1.1 Figures

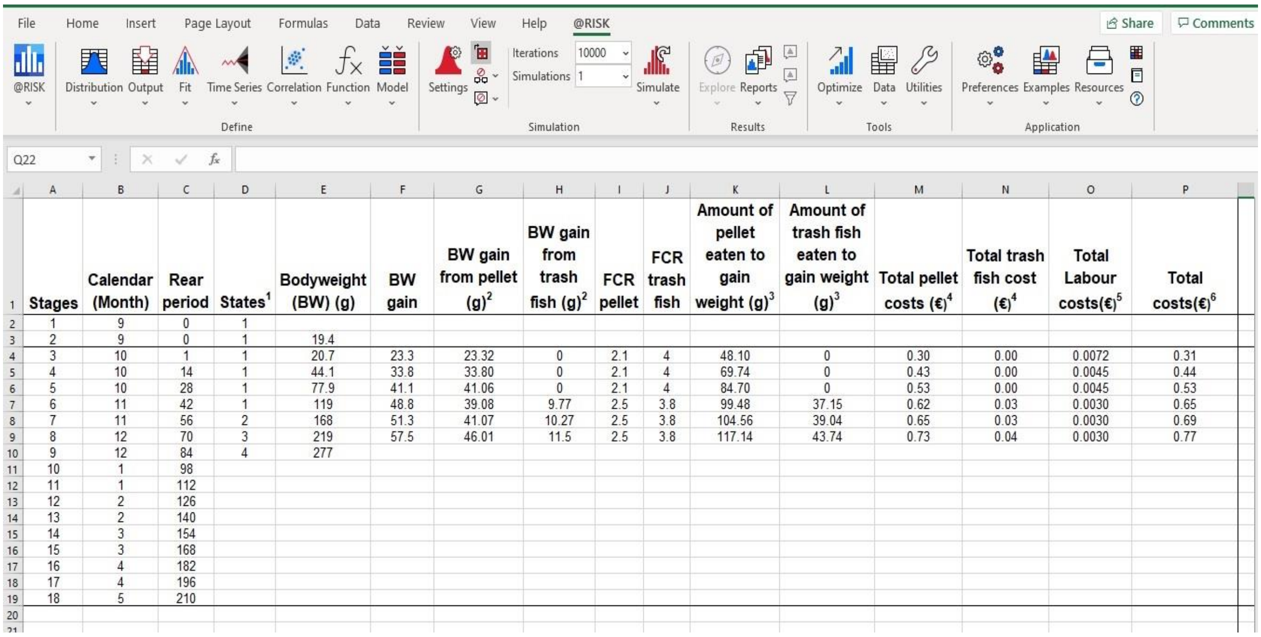

Stages	Calendar (Month)	Rear period	States[1]	Bodyweight (BW) (g)	BW gain	BW gain from pellet (g)[2]	BW gain from trash fish (g)[2]	FCR pellet	FCR trash fish	Amount of pellet eaten to gain weight (g)[3]	Amount of trash fish eaten to gain weight (g)[3]	Total pellet costs (€)[4]	Total trash fish cost (€)[4]	Total Labour costs(€)[5]	Total costs(€)[6]
1	9	0	1												
2	9	0	1	19.4											
3	10	1	1	20.7	23.3	23.32	0	2.1	4	48.10	0	0.30	0.00	0.0072	0.31
4	10	14	1	44.1	33.8	33.80	0	2.1	4	69.74	0	0.43	0.00	0.0045	0.44
5	10	28	1	77.9	41.1	41.06	0	2.1	4	84.70	0	0.53	0.00	0.0045	0.53
6	11	42	1	119	48.8	39.08	9.77	2.5	3.8	99.48	37.15	0.62	0.03	0.0030	0.65
7	11	56	2	168	51.3	41.07	10.27	2.5	3.8	104.56	39.04	0.65	0.03	0.0030	0.69
8	12	70	3	219	57.5	46.01	11.5	2.5	3.8	117.14	43.74	0.73	0.04	0.0030	0.77
9	12	84	4	277											
10	1	98													
11	1	112													
12	2	126													
13	2	140													
14	3	154													
15	3	168													
16	4	182													
17	4	196													
18	5	210													

[1]Stage 1 until stage 6 simulated healthy status of Asian seabass. At stage 7 Asian seabass was simulated to be sub-clinically infected with vibriosis, then in the next stage the state turned into clinical vibriosis. At stage 9, Asian seabass was predicted to dead.
[2]The bodyweight gain from attribution of feed (Eq. 2) is the product of percentage of feed attribution, assuming to be from pellet only (stage 3-5) and later a mix between pellet and trash fish (Refer to Table 4 for the ratio) and bodyweight gain of fish (predicted using a growth function (Eq.3)).
[3]Amount of eaten (Eq. 4) is the product of FCR (Refer to Table 4) of pellet and trash fish with the bodyweight gain attribution of feed.
[4]Total feed costs (pellet and trash fish) was estimated by the product of feed price/kg and amount of feed eaten (Eq. 5-7).
[5]Total labour costs was estimated by the sum of labour cost for grading, cleaning net and feeding (Eq. 8-10).
[6]In the example, the costs of grow-out Asian seabass that die due to vibriosis is the sum of variable costs (total feed cost and total labour cost) to grow-out fish from day 0 post-stocking until dead status (post-stocking day 84).

Figure S1. The example of one iteration in the stochastic bioeconomic model in excel that simulated the cost of infected and dead Asian seabass due to vibriosis. Column A showed stage (1 to 18), column B showed Month (September-May), column C showed rear period from 0 day (hatchery) and 1 to 210 days post-stocking (grow-out), column D showed states of the fish (1=healthy, 2=subclinical vibriosis, 3=clinical vibriosis, 4=dead due to vibriosis) (Figure 3 in main text), column E and F showed bodyweight at each stage, column G and H showed the contribution of weight from feed to estimate amount of feed (column K and L) and total costs of feed (column M and N). The list of equations can be found in Supplementary materials.
