# Supplementary material for: Estimating the Economic Loss Due to Vibriosis in Net-Cage Cultured Asian Seabass (Lates calcarifer): Evidence From the East Coast of Peninsular Malaysia
Source: Front Vet Sci. 2021 Oct 8;8:644009. doi: 10.3389/fvets.2021.644009 (PMC8531722; doi:10.3389/fvets.2021.644009)
Supplement: Supplementary file 2 [file Data_Sheet_2.docx]

Supplementary Material

# Equations used to estimate economic losses of vibriosis using stochastic model

| **Economic losses due to vibriosis**  $=\frac{\boldsymbol{TVdeathV}}{\boldsymbol{NrSurvived}}$  Where;  TVdeathV   = Total variable costs of grow-out fish until dead due to vibriosis (€)  NrSurvived = Number of survived Asian seabass (tail) | **Equation 1** |
| --- | --- |
| **VARIABLE COSTS** |  |
| $\mathbf{Bodyweight gain}\left( \mathbf{g} \right)\mathbf{from attribution of feed}\left( \mathbf{pellet and trash fish} \right)$  $\boldsymbol{=}\boldsymbol{Att\% x BWg}$  Where;  Att_%_  = Percentage of feed attribution  BWg = Bodyweight gain (g per fish per day) | **Equation 2** |
| **Bodyweight gain (g per fish per day) (BWg)**  $=\mathbf{(K + xT + yT}\mathbf{2}\mathbf{+ zT}\mathbf{3}\mathbf{)* (BW)}\mathbf{aT+b}$  Where;  K, x, y, z, a, b = constant  BW      = Geometric mean live bodyweight (g)  T = Temperature (Operational range of 16 – 39 ℃) | **Equation 3** |
| **Amount of feed (pellet and trash fish) eaten (g) (FAMT)**  $=\mathbf{FCR x BWg} \mathbf{Att}$  Where;  FCR          = Feed conversion rate  BWg Att   = Bodyweight gain from attribution of feed (g) | **Equation 4** |
| **Total pellet costs per day (PC)**  $=\mathbf{Pelletp} \mathbf{x PelletAMT}$  Where;  Pelletp         = Pellet price per kg (€)  PelletAMT   = Amount of pellet consumed per day (kg) | **Equation 5** |
| **Total trash fish costs per day (TFC)**  $\boldsymbol{=TFP x TFAMT}$  Where;  TFP            = Trash fish price per kg (€)  TFAMT     = Amount of trash fish consumed per day (kg) | **Equation 6** |
| **Total feed costs (per day) (FC)**  $\boldsymbol{=PC+TFC}$  Where;  PC            = Pellet costs per day (€)  TFC         = Trash fish costs per day (€) | **Equation 7** |
| **Labor costs for grading (GLC)**  $\mathbf{=}\frac{\boldsymbol{Gt x Wage}}{\boldsymbol{Stock Number}}$  Where;  Gt        = Amount of time taken to do grading/cage  Wage   = Labour costs/ seconds  Stock Number   = Stocking density per cage (tail) | **Equation 8** |
| **Labor costs for cleaning net (CLC)**  $\mathbf{=}\frac{\boldsymbol{Ct x Wage}}{\boldsymbol{Stock Number}}$    Where;  Ct          = Amount of time taken to do clean net/cage  Wage    = Labour costs per seconds  Stock Number     = Stocking density per cage (tail) | **Equation 9** |
| **Labor costs for feeding (FLC)**  $\mathbf{=}\frac{\boldsymbol{Ft x Wage}}{\boldsymbol{Stock Number}}$    Where;  Ft     = Amount of time taken to do feeding/cage  Wage   = Labour costs/ seconds  Stock Number   = Stocking density per cage (tail) | **Equation 10** |
| **Total variable costs per tail (VC)**  $\boldsymbol{=FRC+FC+LC}$  Where;  FRC    = Fingerling price per tail (4 inch) (€)  FC      = Total feed costs per tail (€)  LC         = Total labour costs per tail (grading, cleaning net and feeding) (€) | **Equation 11** |
| **FIXED COSTS**  **Petrol cost per tail (PetC)**  $\mathbf{=}\frac{\boldsymbol{PetP for 210 days}}{\boldsymbol{Stock Number}}$    Where;  PetP         = Petrol costs per month x 7 months (€)  Stock_n_ = Stocking density per cage (tail) | **Equation 12** |
| **Maintenance cost per tail (MC)**  $\mathbf{=}\frac{\boldsymbol{MP for 210 days}}{\boldsymbol{Stock Number}}$    Where;  MP = Maintenance costs per month x 7 months (€)  Stock Number = Stocking density per cage (tail) | **Equation 13** |
| **Utility cost per tail (UC)**  $=\frac{\boldsymbol{UP for 210 days}}{\boldsymbol{Stock Number}}$    Where;  UP = Utility costs per month x 7 months (€)  Stock Number = Stocking density per cage (tail) | **Equation 14** |
| **Total fixed costs per tail (FixC)**  $\boldsymbol{=PetC+MC+UC}$  Where;  PetC = Petrol cost per tail (€)  MC = Maintenance cost per tail (€)  UC = Utility cost per tail (tail) | **Equation 15** |
| **PROVISION COSTS**  **Economic losses due to other causes**  $=\frac{\boldsymbol{TVdeathOR}}{\boldsymbol{NrSurvived}}$    Where;  TVdeathOR= Total variable costs of grow-out fish until dead due to other reason (€)  NrSurvived = Number of survived Asian seabass (tail) | **Equation 16** |
| **Total provision costs (PC)**  $\boldsymbol{=TVdeathV+TVdeathOR}$  Where;  TVdeathV = Total variable costs of grow-out fish until dead due to vibriosis (€)  TVdeathOR= Total variable costs of grow-out fish until dead due to other reason (€) | **Equation 17** |
| **PROFIT**  **Total revenue (TR)**  $\boldsymbol{=FishP x BWT}$  Where;  FishP = Fish market price/kg (€)  BWT = BWT (kg) | **Equation 18** |
| **Gross margin (Gross)**  $\boldsymbol{=TR-VC}$  Where;  TR      = Total revenue (€)  VC     = Total variable costs (€) | **Equation 19** |
| **Net Profit (Nett)**  $\boldsymbol{=Gross-FixC}$  Where;  Gross      = Gross margin  FixC     = Fixed costs | **Equation 20** |
| **Total costs of grow-out Asian seabass per tail**  $\boldsymbol{=VC+FixC+PC}$  Where;  VC = Total variable costs per tail (€)  FixC = Total fixed costs per tail (€)  PC = Total provision costs due to mortality per tail (€) | **Equation 21** |
| **Percentage economic losses in production costs of Asian seabass**  $=\frac{Economic losses of vibriosis per tail (€)}{Total costs of grow-out Asian seabass per tail (€)} x 100\%$ | **Equation 22** |
